# Progress towards Health for All: Time to End Discrimination and Marginalization

**DOI:** 10.3390/ijerph17051696

**Published:** 2020-03-05

**Authors:** Stuart Gilmour, Phuong Le Mai, Phuong Nguyen, Bibha Dhungel, Maki Tomizawa, Huy Nguyen

**Affiliations:** 1 Graduate School of Public Health, St. Luke’s Center for Clinical Academia, Susumu & Mieko Memorial, St. Luke’s International University, 3-6-2 Tsukiji, Chuo-ku, Tokyo 104-0045, Japan; lemaiphuong166@gmail.com (P.L.M.); 18mp207@slcn.ac.jp (P.N.); 18mp201@slcn.ac.jp (B.D.); 19mp216@slcn.ac.jp (M.T.); nguyenhuy@slcn.ac.jp (H.N.); 2 Institute for Preventive Medicine and Public Health, Hanoi Medical University, Hanoi 100000, Vietnam

Although it has been more than 40 years since “health for all” was presented as a focus in the Alma Ata declaration [1], the world is still far from achieving this goal. Although great progress has been made since then in many areas of health, much of that progress has been confined to rich countries, and even within the richest of countries, health inequality persists. New health problems and threats have arisen in the generations since that hopeful statement, with the scourge of HIV and the challenge of non-communicable diseases (NCDs) facing high and low-income countries alike [2]. With these new challenges, we see inequalities forming along the same old social fault lines and the same axes of deprivation and discrimination. In high-income countries, new risk factors such as obesity and food insecurity cluster in the poorest communities [3,4], while on a global level, we see the greatest burden of new diseases such as HIV falling on the poorest countries [5], and continuing challenges of health access and financing for the poorest people [6]. Much of this inequality arises from simple economic inequality and the consequences of colonialism, but much also results from the marginalization of certain individuals and groups at community, national and global levels. Sexual minorities, people with mental illness, migrants, refugees, the homeless, transient populations, transgender people, sex workers and many other groups who do not conform to existing social norms, or who have historically been marginalized and excluded, experience many often completely preventable illnesses. Discrimination against some members of the community, their exclusion from economic, cultural and social processes, and the selective provision of basic services to these communities put them at increased risk of poor health and expose them to preventable risk factors. Because the marginalization of these people is socially and historically determined, the health consequences for these people continue to be severe and pernicious, despite being preventable.

In this Special Issue on the health of marginalized people, we have gathered 15 papers from many countries, describing a wide range of physical and mental health issues, using a variety of advanced research methods to understand the health challenges faced by a diverse array of marginalized populations globally, and recommending interventions to improve the health of marginalized populations. First, we summarize the research articles presented in this Special Issue and then issue a call for renewed action to end discrimination and marginalization in our societies, and to strengthen wider efforts to support the health of the most marginalized people. 

Mental health and suicide are significant issues among marginalized populations which do not receive sufficient attention. In this issue, Gilmour et al. [7] describe the suicide mortality rates of foreigners living in Japan compared with those in their country of origin and in Japanese nationals. They showed that, distinct from other nationalities, Koreans living in Japan have substantially higher mortality rates than both Japanese people and Koreans living in Korea, possibly due to the particular social, economic, and cultural pressures that this marginalized population face in Japan. Houser et al. [8] investigated the role that mental health plays in parole decisions in Pennsylvania, in the United States, and found that mental health plays only a limited role in these decisions. This is important evidence for developing guidelines and institutional frameworks for protecting marginalized people from further discrimination in the law enforcement system. 

It is often the poorest who suffer the most during epidemics, but Okland and Mamelund found the opposite during the 1918 Influenza pandemic in the U.S. [9]. The authors’ literature review identified lower morbidity and mortality but a higher case fatality ratio among black Americans compared to whites. Their results suggest that the social conditions of that period led to black Americans having a lower risk of exposure but higher fatality rates after infection, possibly due to high susceptibility to other bacterial diseases, such as pneumonia. This research shows the complex role of social determinants of health in mediating the spread of infectious disease and exacerbating its effects. Another paper in this issue, by Shelley-Egan and Dratwa [10], discusses the global health implications of marginalization in certain countries and communities by analyzing the global health narratives surrounding the 2014–2015 Ebola outbreak in West Africa. Their review described reasons for this health crisis, including the lack of viable policies and responses from local government and international agencies, as well as cultural beliefs and health practices among African people. They showed that marginalization operates at an international as well as a national level, and that such marginalization within the global health community can hinder the response to global health emergencies—an important lesson in a new era of emerging infectious diseases such as COVID-19 [11]. 

As HIV is the canonical example of an infectious disease that thrives in environments of marginalization and discrimination, this special issue naturally included several articles discussing this epidemic. Peng et al. [12] described willingness to use Pre-Exposure Prophylaxis (PrEP) among Men Who Have Sex with Men (MSM) in China and found a high level of willingness to take free PrEP in this high-risk population. They gave suggestions for reducing community and self-stigmatization toward MSM in order to promote PrEP uptake and adherence among this marginalized group. Vendula B. et al. [13] reported on the social needs and acceptance of support among clients of a supervised injecting facility in Australia, where harm reduction is the main response to HIV. They found that people with higher self-perceived need were more likely to accept assistance, and identified referral from low-threshold programs along with increased attention to the improvement of services in high-threshold programs as essential to linking high-risk individuals to appropriate care. Meanwhile, Hill et al. [14] examined condom use and its associated factors among Japanese MSM in Tokyo—another population at high risk of HIV [15]. They showed that inconsistent condom use varies among MSM with different types of sex partners, and the use of condoms is associated with education level, self-efficacy for safer sex, marital status, and drinking alcohol, again showing the role of self-stigmatization and marginalization in increasing risk behaviors. They recommended tailored strategies to improve condom use in this community and show the importance of updating research strategies as well as outreach methods when tackling the HIV epidemic in young, rapidly-changing hidden populations.

The special issue also touches on risk behaviors among marginalized populations. Wang and Shuanglong [16] explored generational differences in smoking, alcohol and dietary consumption behaviours among British minorities. They found that the second generation of migrants is more likely to smoke and consume alcohol and less likely to eat fruits and vegetables than white British, and identified a need for targeting second-generation ethnic minorities adopting unhealthy lifestyles. Pallesen et al. [17] conducted a study of the feasibility of a participatory intervention for improving dietary behaviour among ethnic minority women in Denmark. They reported that minority women of lower socioeconomic backgrounds easily engaged with a culturally relevant and acceptable cardiovascular disease prevention intervention, which positively motivated healthy dietary behaviour, and showed that, with the correct engagement, marginalized groups can have reduced dietary risks. Dearle et al. [18] also explored cultural factors related to risk for NCDs, examining knowledge and attitudes towards diabetes among Fijians. They investigated attitudes towards a traditional diet, the influence of family members, community beliefs, and awareness of diabetes, and found that effective intervention should incorporate cultural values and practices, emphasizing the essential role of family networks and church groups in conveying health messages. 

Many of the studies in this special issue identified strategies to improve the health of marginalized groups. Wallimann and colleagues [19] explored the primary care network of Eritrean immigrants in Switzerland. They found language difficulties and intercultural understanding are barriers to properly accessing care and identified six key lessons for practices to facilitate health care access for Eritrean immigrants. Pudpong et al. [20] assessed a voluntary health insurance scheme for migrants in Thailand. They found that it improved access for previously uninsured migrants and reduced the financial burden for public hospitals in the project area. They suggested further efforts to raise community understanding of the benefits of being insured in order to improve the sustainability and effectiveness of the program. Gaboardi et al. [21] assessed the goals and principles of providers working with people experiencing homelessness in eight European countries, indicating a contrast between providers who were more focused on their client’s autonomy and those whose primary focus was on the client’s basic needs such as food, health and temporary accommodation. Braun-Lewensohn et al. [22] explored the coping strategies for stressors among women in Bedouin society and described the stability of sense of coherence (SOC) in this minority society as it underwent rapid social and cultural changes. Significant differences were found between age groups, and the oldest women were identified as an at-risk population due to limitations in their coping strategies and SOC. In related research in a very different community, Millan et al. [23] examined the health beliefs and practices of Gypsies and Travelers in the UK, where these communities experience significant marginalization and discrimination. They described this community’s health problems and their experiences in health care systems, including discrimination and negative stereotypes. The authors indicate the need for a more concentrated and intensive approach to improve health among travelers in the UK.

The papers in this Special Issue describe a variety of health issues among marginalized communities in both developed and developing nations, ranging from not only infectious to non-communicable diseases but also some emerging problems such as Ebola, HIV, and suicide. Although marginalization remains a significant and serious cause of ill health and health inequality globally, no aspect of the health challenges that marginalized people face is insurmountable. With proper respect for the dignity of every individual and a commitment to end discrimination, marginalization and stigmatization, it is possible to ensure that all members of the global community enjoy the same high standards of health, and by attending to and investing more in the health of marginalized people, we can make progress towards health for all.
